# First hybrid implantations of novel Salus-Valves in patients with severe pulmonary regurgitation: A case series

**DOI:** 10.3389/fcvm.2022.1059664

**Published:** 2022-11-24

**Authors:** Zehua Shao, Shubo Song, Yu Han, Weijie Liang, Taibing Fan

**Affiliations:** ^1^Children’s Heart Center, Zhengzhou University People’s Hospital, Fuwai Central China Cardiovascular Hospital, Henan Provincial People’s Hospital, Zhengzhou, China; ^2^Department of Structural Cardiology, Zhengzhou University People’s Hospital, Fuwai Central China Cardiovascular Hospital, Henan Provincial People’s Hospital, Zhengzhou, China

**Keywords:** pulmonary regurgitation, transcatheter pulmonary valve implantation, right ventricular outflow tract (RVOT), invasive surgical procedures, valve implantation

## Abstract

With the increasing age of patients after right ventricular outflow tract (RVOT) reconstruction, progressive pulmonary valve (PV) dysfunction can result in different degrees of right heart insufficiency, and PV replacement is frequently needed during follow-up. The traditional redo thoracotomy is difficult and associated with higher risks when compared to transcatheter implantations. Herein, we report the advantages and describe the outcomes of the first hybrid implantations of the novel Salus-Valves (Balance Medical, Beijing, China) from the sub-xiphoid approach in five patients (mean age of 22.6 years) with severe pulmonary regurgitation (PR) after RVOT reconstruction.

## Introduction

Pulmonary regurgitation (PR) often occurs after right ventricular outflow tract (RVOT) reconstruction (1). Progressive pulmonary valve (PV) dysfunction can result in different degrees of right heart failure (2, 3). Conventional redo surgery is traumatic and risky. Percutaneous pulmonary valve implantation (PPVI) has been proposed as a less-invasive alternative but cannot be applied in some cases with complex RVOT anatomies and limitations (4). Transthoracic pulmonary valve implantation (TPVI) is another good solution in these conditions. Herein, we report and describe the short-term results of the first hybrid implantations of the novel Salus-Valves (Balance Medical, Beijing, China) from the sub-xiphoid approach.

## Cases report

**FIGURE 1:**
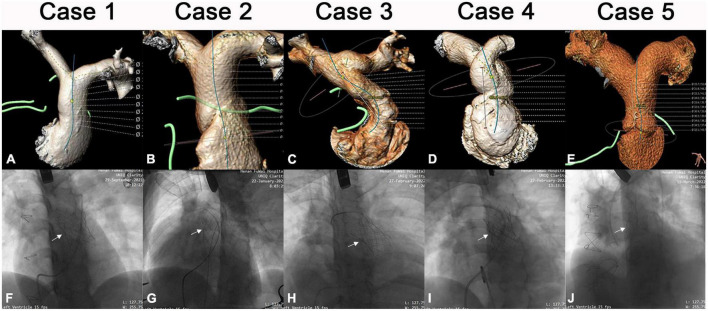
Imaging data of patients 1–5. Pre-operative CTA image of each patient **(A–E)**. Intraoperative image after Salus-valve (white arrows) deployment of each patient **(F–J)**.

Five patients with a mean age of 22.6 years (range, 10–51), diagnosed with severe PR after RVOT transannular patching received hybrid implantations of the novel Salus-Valves *via* the subxiphoid approach at our institution between September 2021 and March 2022. Patients’ demographics and procedural data are outlined in Table 1. All patients had clinical symptoms of chest tightness or shortness of breath. Pre-operative evaluation and procedure planning were done using transthoracic echocardiography (TTE), cardiac computed tomography angiography (CTA), and cardiovascular magnetic resonance imaging (CMR). The pulmonary artery (PA) 3D was anatomy reconstructed based on the CTA image analysis and the narrowest cross-sectional plane and length of the main PA were determined (Figures 1A–E). All patients were discussed at a multidisciplinary conference prior to the procedure. This approach was chosen because patients were deemed higher-risk surgical candidates. All patients signed informed consent for the reported surgical procedures and the publication of clinical data. This study was approved by the Ethical Committee of Central China Fuwai Hospital of Zhengzhou University (ethical No: 2021-Q003-02).

**TABLE 1:** Patients characteristics and follow-up.

	Case 1	Case 2	Case 3	Case 4	Case 5
Age (years)	22	20	10	10	51
Gender	Female	Male	Female	Male	Male
Weight (KG)	56.6	75.2	59.2	29.1	71.3
Diagnosis and surgery	TOF. RVOT reconstruction using transannular patch and VSD repair	TOF. RVOT reconstruction using transannular patch and VSD repair	TOF. RVOT reconstruction using transannular patch and VSD repair	TOF. RVOT reconstruction using transannular patch and VSD repair	VSD with IE Pulmonary valvuloplasty and bioprosthetic mitral and tricuspid valves replacement and VSD repair
Years from corrective surgery	19	16	9	9	13
Pre-operative PV velocity (M/sec)	2.3	1.25	2.74	3.51	2.62
Pre-operative PV regurgitation (cm^2^)	12	9	8.1	7.3	10.4
Pre-operative NYHA	II	III	II	II	IV
Pre-operative RVEDVI	163	175	155	154	165
Pre-operative EF (%)	68	43	69	61	25
RVOT diameter on CTA (mm)	21.1	25	20	18	37
RVOT diameter of balloon interrogation (mm)	25.5	28	22	20 (after balloon dilatation)	30 (after main PA plication)
Balloon size (mm)	34	34	34	34	34
French Access (Fr)	24	24	24	22	24
Salus-Valve size	P-30	P-30	P-24	P-22	P-32
Pulmonary angioplasty before implantation	None	None	None	Transcatheter balloon dilatation	Longitudinal plication of the main PA
Procedure time (min)	131	115	126	132	193
Fluo time (min)	23	21	24	27	31
Drainage days	1	1	1	1	3
Hospitalization days	3	3	3	4	7
FU period (months)	12	8	7	7	6
PO NYHA	I	II	I	I	III
PO EF (%)	73	52	67	67	42
PO RVEDVI (ml/m^2^) (6 months)	93	110	103	93	120
PO PV velocity (M/sec)	2.7	1.6	1.42	2.0	2.2
PO PV regurgitation (cm^2^)	3.2	2.7	2.5	1.1	2.6
Complications	None	None	None	None	None

CTA, cardiac computed tomography angiography; FU, follow-up; IE, infective endocarditis; NYHA, New York Heart Association; PA, pulmonary artery; PV, pulmonary valve; PO, postoperative; RVOT, right ventricular outflow tract; RVEDVI, right ventricular end-diastolic volume index; TOF, Tetralogy of Fallot; VSD, ventricular septal defect.

### Salus-valve

**FIGURE 2:**
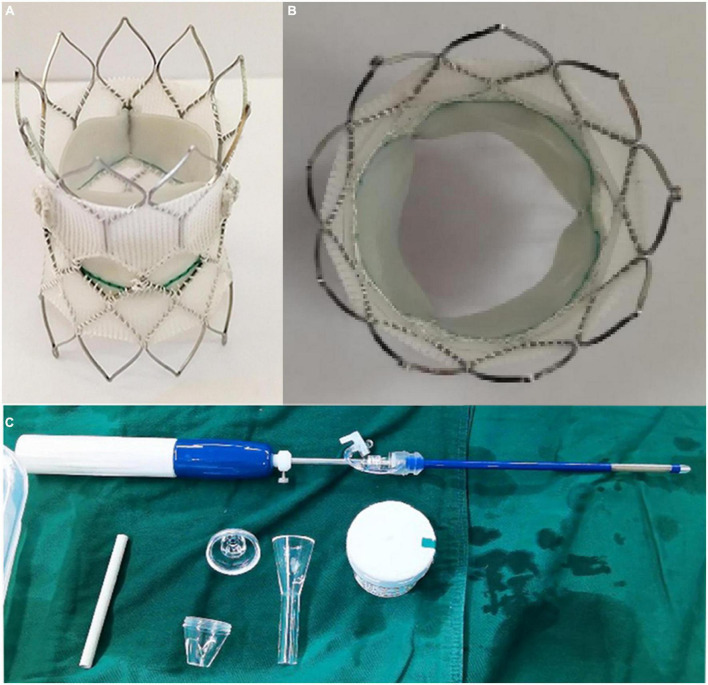
Salus valve **(A,B)** and delivery system **(C)**.

The surgical Salus interventional PV (Balance Medical, Beijing, China) is a bovine pericardial valve sutured to a self-expandable Nitinol stent. The Salus valve is available in 8 diameters (range: 18–32 mm) and can be delivered through 22-Fr for the P18–24 and 24-Fr for the P26–32 (Figure 2). This large portfolio makes the Salus valve suitable for patients with a main PA diameter of 16–34 mm and length ≥ 20 mm.

### Hybrid intervention

**FIGURE 3:**
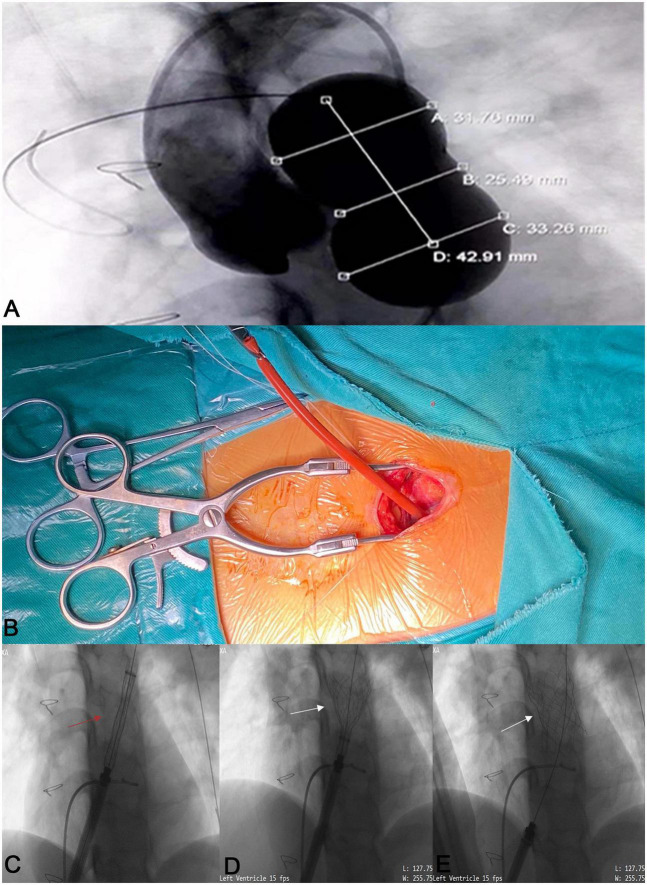
The process of the novel Salus-Valve *via* subxiphoid approach. Intraoperative pulmonary angiography and compliant balloon measurement **(A)**. A 3 cm subxiphoid incision **(B)**. The assembled delivery system (red arrow) was conveyed to the preplanned location **(C)** and release the Salus valve (white arrows) **(D,E)**.

The intervention was carried out in a hybrid operating room. Right-heart catheterization, right ventriculography, and pulmonary arteriography were performed. Taking the CTA analysis results into consideration, RVOT balloon interrogation was done with a simultaneous coronary angiogram to determine the adequate valve size and exclude coronary artery compression (Figure 3A). An incision (about 3 cm) was made beneath the xiphoid process (Figure 3B and Supplementary Video 1). A puncture point was selected at the sharp edge of the right ventricle (RV), and a felt pouch was sewn using a prolene suture (4–0). The wire was inserted through the puncture point and delivered to the Left PA. Five valve sizes (P22–32) were adopted in the five patients based on the balloon interrogation diameter with an additional 2–4 mm. The appropriate valve size and matching delivery sheath were selected, and the system was conveyed along the Guidewire (Cordis Corp., USA) to the selected landing zone (Figures 3C–E and Supplementary Videos 2, 3). All valves were released successfully (Figures 1F–J). To facilitate suitable valve size selection before implantation, patient 4 received transcatheter balloon dilatation of the PV annulus to treat PV stenosis and patient 5 had longitudinal plication of the main PA through a small parasternal incision to treat severe dilation of the PA.

### Outcomes

Following implantation, there was no PV regurgitation, valve displacement, coronary artery compression, and reduction of blood flow in the PA branches (Supplementary Video 4). On 1-month follow-up, TTE revealed no PV regurgitation or paravalvular leakage. At 6 months of follow-up, RV end-diastolic volume index declined from 162.40 ± 8.532 ml/m^2^ to 104.20 ± 11.122 ml/m^2^ (*p* < 0.05), and all patients reported clinical improvement with an increase by one grade in NYHA classes.

## Discussion

Patients with complex congenital heart diseases and RVOT obstruction usually undergo RVOT reconstruction at an early stage (5). In Western countries, valved conduits are more commonly used to reconstruct the RVOT. In China, RVOT reconstruction is usually performed with PA transvalvular ring patch. During follow-up, these patients will show progressive RVOT dysfunction and may need to undergo multiple valve replacements in their lifetime. Redo thoracotomy is more difficult and associated with higher risk and complications, and thereby PPVI has gradually become the most preferred treatment method for these patients (6–8).

The two most commonly used PPVI systems are the balloon-expandable Melody valve (Medtronic Inc., USA) and the Sapien valve (Edwards Lifesciences, USA). The Melody valve is suitable for RVOT diameters smaller than 24 mm, and the maximum size of the Sapien valve is 29 mm (9–11). Many patients were not able to receive these valves due to large RVOT diameters and irregular postoperative RVOT anatomies (4, 12). Newer PPVI systems such as Venus P-valve (Venus MedTech, Hangzhou, China) and Myval valve (Meril LifeSciences Pvt., Ltd., India) have been used to treat these patients (13–15). Until now, the Venus P-valve has the largest diameter of all PPVI systems. The Venus P-valve is composed of a self-expanding Nitinol framework with a tri-leaflet porcine pericardial valve in both straight and flared designs. The diameters of both designs ranged from 18 to 36 mm with increments of 2 mm (16). The Venus P-valve has been implanted in many countries and clinical results are good (13, 14, 17). However, in some countries, the device has not been approved for clinical use by regulatory bodies. In addition, we have observed that even the largest Venus P-valve is still not suitable for patients with extra-large RVOTs, such as in patient No. 5. The manipulation of the large and extra-large delivery systems through the classical transvenous route can be extremely challenging even in patients with RVOT diameters within the range of currently available PPVI systems.

For all those aforementioned reasons, we selected and used the Salus PV and initiated this new program of hybrid implantation *via* the subxiphoid approach. Compared with traditional surgical PV replacement, this approach is less invasive, less aggressive, and has a strong potential in shortening the postoperative recovery time. Compared with PPVI, this approach is not limited by the condition of peripheral vascular access and the sheath diameter and thereby can be applied to younger patients with small or even occluded femoral veins. This approach also offers the advantages of the short direct path with easier control of the PV delivery and implantation, while avoiding any damage to the tricuspid valve. The hybrid setting of the procedure also offers the possibility of a small incision pulmonary valvuloplasty or percutaneous pulmonary valvuloplasty when needed in wide and twisted PA to select the best valve size.

In the present study, the novel Salus PV *via* subxiphoid approach was adopted for the treatment of severe PR after RVOT reconstruction and the short-term results are satisfactory. We present a new hybrid approach that is feasible, safe, minimally invasive, and worth popularizing in clinical practice after addressing the limitations of this case series in large well-conducted studies with longer follow-ups.

## Data availability statement

The raw data supporting the conclusions of this article will be made available by the authors, without undue reservation.

## Ethics statement

The studies involving human participants were reviewed and approved by the Ethical Committee of Central China Fuwai Hospital of Zhengzhou University (ethical No: 2021-Q003-02). Written informed consent to participate in this study was provided by the participants or their legal guardian/next of kin. Written informed consent was obtained from the individual(s), and minor(s)’ legal guardian/next of kin, for the publication of any potentially identifiable images or data included in this article.

## Author contributions

ZS helped design the project, helped invent the surgical method, and was responsible for collecting data and writing the manuscript. SS participated in the operation and data collection and assisted in the writing. YH and WL were part of the operation and part of the data collection. TF was responsible for the design of subjects and the invention of surgical methods. All authors contributed to the article and approved the submitted version.
